# Supplementary material for: Biogas slurry application alters soil properties, reshapes the soil microbial community, and alleviates root rot of Panax notoginseng
Source: PeerJ. 2022 Jul 26;10:e13770. doi: 10.7717/peerj.13770 (PMC9336633; doi:10.7717/peerj.13770)
Supplement: Supplemental Information 1 — Pairwise Groups: The two groups applied for assessing statistical significance of differences in bacterial or fungal composition among different pairwise groups using PERMANOVA (Bray–Curtis, permutation = 999); R2: Interpretation degree of difference; P value and Significance: Reliability of the statistical test (P value), “-” indicates P ≥ 0.05, “*” indicates 0.01 P < 0.05, “**” indicates 0.001 < P ≤ 0.01, and “***” indicates P ≤ 0.001. [file peerj-10-13770-s001.doc]

|  | Pairwise Groups | R^2^ | *P* value | Significance |
| --- | --- | --- | --- | --- |
| Bacteria | Soil vs. CHCF0d | 0.94 | 0.016 | * |
|  | Soil vs. CK15.30d | 0.45 | 0.062 | - |
|  | Soil vs. CH15.30d | 0.64 | 0.018 | * |
|  | Soil vs. CF15.30d | 0.71 | 0.013 | * |
|  | CHCF0d vs. CK15.30d | 0.88 | 0.003 | ** |
|  | CHCF0d vs. CH15.30d | 0.71 | 0.002 | ** |
|  | CHCF0d vs. CF15.30d | 0.70 | 0.001 | *** |
|  | CK15.30d vs. CH15.30d | 0.66 | 0.001 | *** |
|  | CK15.30d vs. CF15.30d | 0.72 | 0.004 | ** |
|  | CH15.30d vs. CF15.30d | 0.28 | 0.002 | ** |
| Fungi | Soil vs. CHCF0d | 0.94 | 0.009 | ** |
|  | Soil vs. CK15.30d | 0.27 | 0.053 | - |
|  | Soil vs. CH15.30d | 0.51 | 0.022 | * |
|  | Soil vs. CF15.30d | 0.55 | 0.01 | ** |
|  | CHCF0d vs. CK15.30d | 0.87 | 0.001 | *** |
|  | CHCF0d vs. CH15.30d | 0.66 | 0.002 | ** |
|  | CHCF0d vs. CF15.30d | 0.66 | 0.001 | *** |
|  | CK15.30d vs. CH15.30d | 0.53 | 0.003 | ** |
|  | CK15.30d vs. CF15.30d | 0.55 | 0.004 | ** |
|  | CH15.30d vs. CF15.30d | 0.34 | 0.001 | *** |
